# Phytochemical, Cytotoxicity, and Antimycobacterial Activity Evaluation of Extracts and Compounds from the Stem Bark of *Albizia coriaria* Welw ex. Oliver

**DOI:** 10.1155/2022/7148511

**Published:** 2022-01-22

**Authors:** Samuel Baker Obakiro, Ambrose Kiprop, Isaac K'owino, Moses Andima, Richard Oriko Owor, Robi Chacha, Elizabeth Kigondu

**Affiliations:** ^1^Department of Pharmacology and Therapeutics, Faculty of Health Sciences, Busitema University, P.O. Box 1460, Mbale, Uganda; ^2^Department of Chemistry and Biochemistry, School of Sciences and Aerospace Studies, Moi University, P.O. Box 3900-30100, Eldoret, Kenya; ^3^Africa Centre of Excellence II in Phytochemicals, Textile and Renewable Energy (ACE II PTRE), Moi University, P.O. Box 3900-30100, Eldoret, Kenya; ^4^Department of Pure and Applied Chemistry, Faculty of Science, Masinde-Muliro University of Science and Technology, P.O. Box 190-50100, Kakamega, Kenya; ^5^Department of Chemistry, Faculty of Science Education, Busitema University, P.O Box 236, Tororo, Uganda; ^6^Centre of Respiratory Disease Research, Kenya Medical Research Institute, P.O. Box 54840-00200, Nairobi, Kenya; ^7^Centre of Traditional Medicine and Drug Research, Kenya Medical Research Institute, P.O. Box 54840-00200, Nairobi, Kenya

## Abstract

**Background:**

*Albizia coriaria* Welw ex. Oliver (Fabaceae) is one of the plants used by herbalists in the East Africa community to prepare herbal remedies for the management of symptoms of TB. Despite its widespread use, the antimycobacterial activity of this plant was uninvestigated and there was contradicting information regarding its cytotoxicity.

**Methods:**

Cytotoxicity (MTT), antimycobacterial activity (MABA), and phytochemical screening were conducted on crude extracts (hexane, chloroform, acetone, and methanol) of the stem bark of *A. coriaria*. Gas chromatography-mass spectrometry (GC-MS) followed by Fourier transform infrared (FTIR) spectroscopy was carried out on the acetone and methanol extracts. The binding affinities and descriptors of pharmacokinetics and toxicity of the identified compounds were predicted using computational modelling software.

**Results:**

The cytotoxic concentrations of all extracts were greater than 1000 *μ*g/mL. The minimum inhibitory concentration of both the acetone and methanol extracts was 1250.0 ± 0.0 *μ*g/mL against *M. smegmatis,* whereas that against *M. tuberculosis* was 937.0 ± 442.0 *μ*g/mL and 2500.0 ± 0.0 *μ*g/mL, respectively. Hexane and chloroform extracts were not active against both strains. Alkaloids, triterpenes, flavonoids, tannins, and saponins were the predominant phytochemicals present. GC-MS analysis revealed twenty-eight and nineteen compounds in acetone and methanol extracts, respectively. Among these was hydroquinone, which was previously reported to possess antimycobacterial activity. Seven compounds identified through GC-MS analysis had better binding affinities for the mycobacterial ATPase and polyketide synthase-13 than isoniazid and rifampicin. These compounds also showed variable but promising pharmacokinetic properties with minimum toxicity.

**Conclusion:**

There are phytochemicals in *A. coriaria* stem bark with potential antimycobacterial activity and acceptable cytotoxicity, which can be further explored and optimized for the development of novel antitubercular drugs.

## 1. Introduction

Tuberculosis (TB) is among the top ten diseases with high mortality and morbidity worldwide [1]. TB is caused by *Mycobacterium tuberculosis* (*M. tuberculosis*), a highly infectious bacillus that mainly affects the pulmonary system but also other extrapulmonary organs [2]. In 2020, 1.5 million deaths of TB were recorded worldwide, of which Africa and South-East Asia contributed 85% [1]. Uganda, Kenya, and the United Republic of Tanzania are among the thirty most burdened countries by TB worldwide [1]. Over the past decades, tremendous success has been achieved in the management of TB. However, the rapid emergence and spread of multidrug-resistant *M. tuberculosis* strains have reduced the efficacy of the first-line antitubercular drugs resulting in treatment failures [3]. Additionally, the current antitubercular drugs are administered for a long time (6–9 months), potentially interact with antiretroviral drugs, and are also associated with several side effects that reduce adherence to treatment [4, 5]. Hence, there is a need to identify new efficacious and safe antitubercular drugs with shorter periods of administration.

Medicinal plants continue to be a leading source of novel molecules for the development of new therapies against various ailments [6]. *Albizia coriaria* Welw ex. Oliver (Fabaceae) is one of the widely used plants in the preparation of herbal remedies for the management of symptoms of TB in the East Africa community [7–10]. Some of the common names of *A. coriaria* include worm-bark, cherry-blossom, false-thorn, worm-cure Albizia tree, (English), *Ober*, *Omogi* (Luo), *Olerai* (Masai), *Musengertet* (Nandi), and *Omubele* (Luhya) in Kenya, *Itek*, *Bata* (Lango), *Ober*, *Ayekayek* (Acholi), *Musita* (Lusoga), *Mugavu* (Luganda), *Etek*, *Etekwa* (Ateso), *Musiisa* (Lukiga and Lutoro), and *Murongo* (Lunyankore) in Uganda [11]. *A. coriaria* is a deciduous tree that grows up to 35 m tall with fewer spreading branches. The stem bark is rough, grey-black, and raggedly scaling. The young branches are hairy with alternate leaves, bipinnately compound with 3–7 pairs of pinnae, oblong to elliptic, wide, and rounded. The flowers are whitish-red, scented with hanging red stamen bearing half-spherical anther heads. The fruit is a brown pod with a pointed apex [12]. *A. coriaria* is less geographically distributed occurring in Côte d'Ivoire, Ethiopia, Uganda, Kenya, Tanzania, Zambia, and northern Angola [11].

Apart from its use in the management of TB, the stem bark of this tree is also used in the preparation of herbal remedies for the management of symptoms of bronchial infections, dysentery, malaria, jaundice, and skin infections [7, 8, 10]. The ethanol extract from the leaves of *A. coriaria* showed good antibacterial activity against several bacteria strains (*Escherichia coli*, *Staphylococcus aureus, Pseudomonas aeruginosa,* and *Salmonella typhi*) with minimum inhibitory concentrations ranging between 62.5 and 250 *μ*g/mL [13]. However, there was no published scientific study that has validated the claimed antimycobacterial activity of *A. coriaria*. Therefore, this study was conducted to determine the cytotoxicity, *in vitro* antimycobacterial activity, and phytochemical composition of the crude extracts of the stem bark of *A. coriaria*. The binding affinities for selected molecular targets of *M. tuberculosis* together with the descriptors of pharmacokinetic and toxicity properties of the identified compounds from GC-MS analysis were also determined using *in silico* studies.

## 2. Materials and Methods

### 2.1. Plant Sample Collection and Preparation

Samples of the stem bark of *A. coriaria* were harvested from trees growing in the wild in Siaya and Kisumu counties, Western Kenya, after identification by a taxonomist. The samples were packed in a clean sack and transported to the Chemistry Laboratory, Moi University, for drying and processing. A voucher specimen (OSB/01/2020/002) was prepared and deposited at the Herbarium, Botany Department, University of Eldoret (Kenya), for authentication and reference purposes. The stem barks were chopped into small pieces and dried under shade at room temperature (25.0 ± 2.0°C) for one month until a constant mass was obtained. The dried samples were then ground using an electric grinder (NutriBullet® 600 Series), packed, and stored in clean labeled paper envelopes at room temperature until extraction.

### 2.2. Extraction

Solvents of analytical grade were purchased from Merck-Sigma-Aldrich and used to prepare crude extracts. Serial extraction was performed using solvents of increasing polarity in the order *n-*hexane, chloroform, acetone, and methanol. The pulverized sample (300 g) was soaked in 1000 mL of hexane for 72 hours with occasional shaking using a digital shaker. The mixture was then filtered through Whatman No. 1 filter paper to obtain the hexane crude extract. The *n-*hexane extract was concentrated using a rotary evaporator (Hahnvapor HS-2005S) at 40°C and reduced pressure. The concentrated crude extracts were dried in a desiccator over anhydrous copper (II) sulfate to constant weight at room temperature. The percentage yield (%) of the extracts was calculated using the following equation:

(1) $$\begin{array}{c} \text{percentage yield}=\frac{\text{weight of dry crude extract}}{\text{weight of the powder macerated}}\times 100. \end{array}$$

Meanwhile, the plant residue was air-dried for 48 hours at room temperature and then remacerated in 1000 mL of the next solvent (chloroform) at room temperature for 72 hours with intermittent shaking and the process of filtration and re-extraction was repeated until the final solvent (methanol). The four concentrated crude extracts were stored in clean labeled bottles at 4°C in a refrigerator until further use.

### 2.3. Cytotoxicity Evaluation of the Plant Extracts

The 3-[4, 5-dimethylthiazol-2-yl]-2, 5 diphenyl tetrazolium bromide (MTT) assay was used to assess the cytotoxicity of the extracts using mammalian Vero E6 cells (ATCC CCL 81) [14]. Cryopreserved Vero E6 cells were revived by incubating them in T_175_ cell culture plates containing growth media at 37°C, 5% carbon dioxide supply, and 98% humidity for 24 hours. The cells were trypsinized and regrown until the required seeding density (20,000 cells/100 *μ*L) was obtained. Vero cells were seeded in a 96 well plate at 2 × 10^4^ cells per well in columns 1, 2, 4, 5, 7, 8, 10, and 11 in Eagle's minimum essential media (MEM) supplemented with 10% fetal calf serum, 1% penicillin/streptomycin, 1% glutamine, and 2.5% of 7.5% (w/v) sodium bicarbonate solution. Wells in rows 3, 6, 9, and 12 received only growth media and served as test extract blank. The cells were grown in 5% carbon dioxide at 37°C for 24 hours. The growth media were removed and replaced with 100 *μ*l of maintenance media (MM). To make initial 1000 *μ*g/mL for serial tripling dilution, a 15 *μ*l of 10 mg/ml of each extract was added to row H of 96-well plates followed by 35 *μ*L of MM and serial tripling dilutions carried out up to row B, representing 1000.00, 333.33, 111.11, 37.04, 12.35, 4.12, and 1.37 *μ*g/mL, leaving row A to serve as cell control. The last 50 *μ*L picked from row B was discarded. Rifampicin was used as a reference standard drug. For each test sample, two columns were used as a negative control (media and plant extract only). Cells were then incubated at 37°C for 48 hours after which the wells were emptied of the media. MTT (10 *μ*L prepared at 5 mg/mL in phosphate-buffered saline) was added to all the wells and incubated for 4 hours until a purple precipitate was visible in the wells under the light microscope. Clear wells were cell-free wells or wells where cells were destroyed by the cytotoxic effect of extracts. The liquid content of the wells was aspirated off, and 100 *μ*L of DMSO was added to dissolve the formazan crystals (attached to the wells) produced by viable cells. The absorbance was read using an ELISA plate reader at 540 nm with a reference wavelength of 720 nm. The percentage cell viability at different extracts' concentrations was calculated using the following equation:

(2) $$\begin{array}{c} \text{percentage cell viability}=\frac{At-Ab}{Ac-Ab}\times 100, \end{array}$$

where *At* is the absorbance value of the test compound, *Ab* is the absorbance value of the negative control (blank), and *Ac* is the absorbance value of the positive control.

The results were used to construct graphs of percentage cell viability against extract concentrations. The cytotoxic concentration of plant extract, which corresponds to 50% viability of the Vero cells (CC_50_ value), was estimated from the graph using a linear regression equation (*Y* = *aX* + *b*).

### 2.4. Antimycobacterial Testing

#### 2.4.1. Mycobacterial Strains and Preparation of Inoculum

Fresh middle brook 7H9 broth media supplemented with glycerol and OACD was prepared according to the manufacturer's instructions. Preserved strains of *M. tuberculosis* (H37R_v_) and *Mycobacterium smegmatis* (ATCC 607) were obtained from the TB laboratory at the Centre for Respiratory Disease Research (CRDR), Kenya Medical Research Institute, Nairobi, Kenya. *M. smegmatis* was used because of its nonpathogenicity, first replicating nature and genetic similarity to *M. tuberculosis* [15, 16]. The obtained seed stock was thawed from the frozen state (−20°C) to room temperature and diluted in a ratio of 1 : 1000 (v/v) with filter-sterilized broth media in a 750 mL culture bottle. The bacteria were precultured for 48 hours for *M. smegmatis* and 14 days for *M. tuberculosis* at 37°C with no shaking up to an OD_600_ 0.6–0.8 (exponential phase) in filter-sterilized 7H9 media supplemented with 10% OADC, 0.2% glycerol, and 0.25% Tween 80 (20% in H_2_O). The pregrown inoculum was again diluted in a ratio of 1 : 1000 at the time of use [17].

#### 2.4.2. Preparation of Plant Extract Stock Solution

Each crude solvent extract (100 mg) was dissolved in 1 mL of dimethyl sulfoxide (DMSO) and vortexed for fifteen (15) minutes. Freshly prepared middle brook 7H9 broth media (9 mL) was then added to the mixture to make a 10,000 *μ*g/mL stock solution with 1% DMSO. The solution was then filter-sterilized (0.22 *μ*m) to obtain an extract stock solution (5 mL). Each extract was prepared fresh at the time of use.

#### 2.4.3. Broth Microdilution Assay

Microplate Alamar blue assay (MABA) was used to determine the minimum inhibitory concentration (MIC) of the extracts in a 96- well plate twofold dilution setup [18]. The broth media 7H9 (50 *μ*L) were added in all the wells, except the first column where the initial concentration of plant extracts was added. Each plant extract was pipetted in duplicate (100 *μ*L of stock solution in column 1), with two rows (1st and 8th) serving as negative control and positive control. Twofold serial dilution was carried out by transferring 50 *μ*L of the content (plant extract + broth media) of the wells of the first column to the next wells until the wells of the 12th column were reached, where 50 *μ*L was aspired off. An aliquot (50 *μ*L) of the pregrown inoculum (*M. smegmatis and M. tuberculosis*) was separately added in all wells except the row 1 wells serving as the control. The total volume in each well was 100 *μ*L, and the initial concentration was diluted such that the concentration of the tested extracts ranged between 5000 and 4 *μ*g/mL. After sealing the microplates with parafilm paper, they were placed in a tight box and incubated for 48 hours for *M. smegmatis* and 21 days for *M. tuberculosis* at 37°C at the biosafety level-3 laboratory at CRDR, KEMRI. Freshly prepared resazurin blue dye (20 *μ*L) at 0.01% in distilled water was added, and the plates were incubated again for another 24 hours. The change in colour from blue to pink indicated the growth of mycobacterium (inactivity of the plant extract) and the noncolour change indicated the inhibition of growth of mycobacterium (the activity of the plant extract). Rifampicin was used as the reference standard drug in this study. The MIC of each extract was determined as the lowest extract concentration that prevented a colour change from blue to pink. Each extract was tested in duplicate, and the mean value was calculated using Microsoft Excel.

### 2.5. Phytochemical Screening

Preliminary qualitative tests were conducted to determine the presence of different phytochemicals in the extract mainly saponins, alkaloids, triterpenes, tannins, flavones, coumarins, reducing sugars, steroid glycosides, emodols, anthocyanins, and volatile oils in the extracts following standard procedures [19].

### 2.6. Gas Chromatography-Mass Spectrometry (GC-MS) Analysis

#### 2.6.1. Sample Preparation

The crude acetone and methanol that showed the highest bioactivity against the mycobacterial species were subjected to GC-MS analysis. Each extract (5 mg) was redissolved in their respective analytical grade solvents (5 mL) and shaken using a digital shaker to ensure complete dissolution. A combination of trimethylchlorosilane (TMCS) and N,O-bis(trimethylsilyl)-trifluoroacetamide (BSTFA) was used to derivatize the alcohols, alkaloids, amines, biogenic amines, steroids, phenols, and carboxylic acids that could be present in the samples for increased detection by the GC-MS/MS system. Excess BSTFA+ 1% TMSC (20 mL) was added to dissolved samples and heated at 70°C for 30 minutes to allow complete derivatization as per the manufacturer's instructions.

#### 2.6.2. GC-MS Sample Analysis

The derivatized samples were analyzed using an Agilent Intuvo 9000 GC system (USA) equipped with a fused silica column (Agilent Intuvo HP-5MS UI) with dimensions 30 m × 250 *μ*m × 0.25 *μ*m. The MS component was Agilent 7000D GC/TQ. Ultrapure helium gas (99.9995%) was used as the carrier gas at a constant flow rate of 1.1 mL/min. An electron ionization energy method with high ionization energy of 70 eV was used for spectral detection with 100 ms of scan time and fragments ranging from 50 to 600 *m*/*z*. An injection volume of 1 *μ*L was used (split ratio 10 : 1), and the injector temperature was maintained at 280°C (constant). The column oven temperature was set at 70°C for 2 minutes, raised at 25°C per minutes up to 150°C, raised at 3°C per minute up to 200°C, raised at 8°C per minute up to 280°C, and held at 280°C for 10 minutes. The contents of phytochemicals present in the test samples were identified based on a comparison of their retention times and mass spectral patterns with those spectral databases of authentic compounds stored in the National Institute of Standards and Technology (NIST) library [20]. The identified compounds were then subjected to an extensive literature search in online databases (PubMed, Scopus, Google Scholar, PubChem, and Web of Science) to assess whether there has been any antimycobacterial or antibacterial activity that has been reported about them.

### 2.7. Fourier Transform Infrared (FTIR) Spectroscopic Analysis

FTIR spectra were acquired using a JASCO FTIR-6600 spectrometer (Japan) to determine the functional groups present in the extracts. Solid samples (1 mg) of each extract were placed on the sample holder of the FTIR spectrophotometer and scanned between 4000 and 400 cm^−1^ to obtain the spectra that were then interpreted.

### 2.8. *In Silico* Studies

Molecular operating environment (MOE) software, version 2008.10 (Chemical Computing Group, Montreal, Canada) was used to perform *in silico* docking simulations. Default settings in the MOE docking simulation tool were used for docking using the London Gibbs free energy scoring function. Before docking simulations were performed, ligands and proteins were prepared, and the docking method was validated.

### 2.9. Ligand Preparation

A local library of compounds from *A. coriaria* identified by GC-MS was prepared for *in silico* studies. Two-dimensional structures of the molecules (ligands) were drawn using ChemBioDraw ultra software, version 12.0 (Cambridge soft), and imported into the MOE structure builder tool as SMILES string to generate 3D structures. The energy of the 3D molecules was minimized by applying the Hamiltonian MMFF94X force field. The minimized molecules were used to develop a local library for docking simulations.

### 2.10. Protein Preparation

The crystal structures of two key molecular targets involved in TB disease were downloaded from the protein data bank (PDB). These were polyketide synthase-13 pks13 (PDB ID: 5V3Y) and ATPase (PDB ID: 3AR4). The proteins were prepared using the LigX tool in the MOE suite. Partial charges were added, the amino acids were protonated, and the crystal structures were sequentially minimized in three stages using the minimization protocol with default parameters. Finally, all counter ions and water molecules were deleted, and the binding sites were defined based on the cocrystalized ligands.

### 2.11. Docking Method Validation

The docking method was first validated by self-docking the native ligand into the binding site of the protein. Here, an induced-fit docking mode was adopted (where both the ligand and the amino acid side chains were left flexible to achieve an optimal fit). The validity of the docking method was evaluated based on the root mean square deviation (RMSD) upon superposition of the best-docked pose to the experimental native ligand conformation. The RMSD value <2 Å was considered optimal. The binding affinities measured in terms of S-score in Kcal/mol were used for comparison with the binding affinities of the test ligands (identified compounds).

### 2.12. *In Silico* Docking of the Compound Library

The local library of the identified compounds was docked into the binding site of the target proteins. The resultant docking poses of the ligands were ranked based on the S-score (binding energy). In this case, the more negative the S-score, the stronger the binding affinity to the target protein. The binding affinities were compared with those of standard drugs used in TB management (isoniazid, rifampicin, and bedaquiline).

### 2.13. Prediction of the Drug-like Properties of the Identified Compounds

To calculate the absorption, distribution, metabolism, elimination, and toxicity (ADMET) properties of the identified compounds from *A. coriaria*, the open-source tools SwissADME (http://www.swissadme.ch/) and ADMETLab 2.0 from computational Biology & Drug Design Group (https://admetmesh.scbdd.com/) were used [21]. This software is freely available online and robustly offers up-to-date and high-quality data. ADMETLab 2.0 uses Caco2-cell (heterogeneous human epithelial colorectal adenocarcinoma cell lines) and MDCK (Madin–Darby Canine Kidney) cell models to predict oral drug absorption, skin permeability, human intestinal absorption, and transdermal drug absorption. Similarly, the program uses blood-brain barrier (BBB) penetration and plasma protein binding models to predict the distribution of the compounds. The test molecules were input as SMILES, and the properties were calculated using the algorithms [22].

### 2.14. Statistical Analysis

Numerical data were entered into Microsoft Excel (version 2013), and the means and standard deviations were calculated. Statistically significant differences were determined using one-way analysis of variance (ANOVA) and/or Student's *t-*test followed by Dunnett's post hoc test using GraphPad Prism (version 5.01, GraphPad Software, San Diego, California, USA). The results were presented as means ± standard deviations, while statistically significant differences were considered at *p* < 0.05.

## 3. Results

### 3.1. Extraction Yield, Antimycobacterial Activity, and Cytotoxicity

Methanol had the highest extraction yield (23.2%) followed by acetone (23.0%), chloroform (0.73%), and lastly hexane (0.48%). All four extracts of *A. coriaria* had cytotoxic concentrations greater than 1000 *μ*g/mL against Vero E6 cells. When compared to rifampicin (CC_50_ = 520.02 ± 40.11), all the other extracts were relatively safer with statistically significant cytotoxic concentrations (Table 1). Acetonic and methanolic extracts of *A. coriaria* had similar antimycobacterial activity (MIC = 1250.0 ± 0.0 *μ*g/mL) against *M. smegmatis,* whereas the acetonic extract was more active (MIC of 937.0 ± 442.0 *μ*g/mL) than the methanolic extract (MIC = 2500 ± 0.0 *μ*g/mL) against *M. tuberculosis* (Table 1). Both the hexane and chloroform extracts were inactive against both *M. tuberculosis* and *M. smegmatis*. Rifampicin had superior activity than the four extracts against the two mycobacterial strains (*p* < 0.05).

### 3.2. Phytochemical Screening Results

The most abundant phytochemicals were tannins, triterpenes, flavonoids, alkaloids, anthocyanins, saponins, steroid glycosides, and coumarins. Emodols were absent in all the extracts, whereas anthracenosides and volatile oils were absent in the methanol extracts. The acetone extract had the highest phytochemical diversity followed by methanol extract, chloroform extract, and lastly hexane extract (Table 2).

### 3.3. GC-MS Results

**Figure 1:**
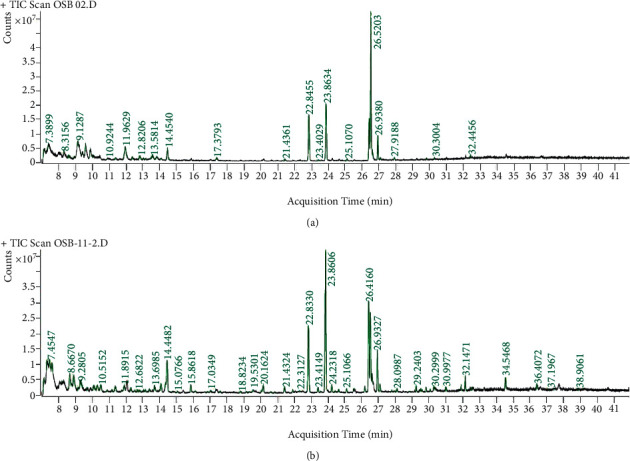
Chromatogram for the GC-MS/MS analysis of the crude acetone (a) and methanol (b) extract of *Albizia coriaria.* Acquisition parameters: oven, initial temp 70°C for 2 min, ramp 25°C/min to 150°C, 3°C/min to 200°C, 8°C/min to 280°C hold for 10 minutes. Injection auto 280°C, volume = 1 *μ*L; split = 10 : 1; carrier gas = He; transfer temp 280°C; source temp 280°C; scan 50–600 Da; column: 30m × 250 *μ*m × 0.25 *μ*m.

**Figure 2:**
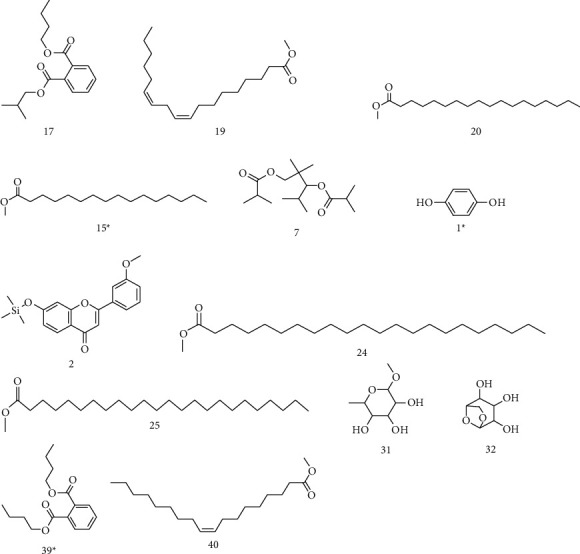
Chemical structures of the most abundant and bioactive∗ compounds identified by the GC-MS analysis in the acetonic and methanolic extract of *Albizia coriaria*.

The GC-MS chromatograms of the acetonic and methanolic extracts revealed a total of 28 and 19 compounds, respectively, that corresponded to the authentic compounds in the NIST library (Table 3 and Figure 1). The most abundant compounds in the acetonic extract included 1,2-benzenedicarboxylic acid, butyl 2-methylpropyl ester (**17**), 9,12-octadecadienoic acid (Z,Z)-, methyl ester (**19**), methyl stearate (**20**), hexadecanoic acid, methyl ester (**15**), 2,2,4-trimethyl-1,3-pentanedioldiisobutyrate(**7**), hydroquinone(**1**), 7-hydroxy-3′-methoxyflavone, trimethylsilyl ether (**2**), docosanoic acid, methyl ester(**24**), tetracosanoic acid, and methyl ester (**25**) (Figure 2). The most abundant compounds in the methanolic extract were hexadecanoic acid, methyl ester (**15**), 2H-pyran-3,4,5-triol,tetrahydro-2-methoxy-6-methyl-(**31**),beta-D-glucopyranose,1,6-anhydro-(**33**), dibutyl phthalate (**39**), 9-octadecenoic acid (Z)-, methyl ester (**40**), and methyl stearate (**20**). Of all the identified compounds, three compounds [hydroquinone (**1**), hexadecanoic acid, methyl ester (**15**), and dibutyl phthalate (**39**)] have been reported to possess antibacterial activity (Figure 2).

### 3.4. FTIR Analysis

**Figure 3:**
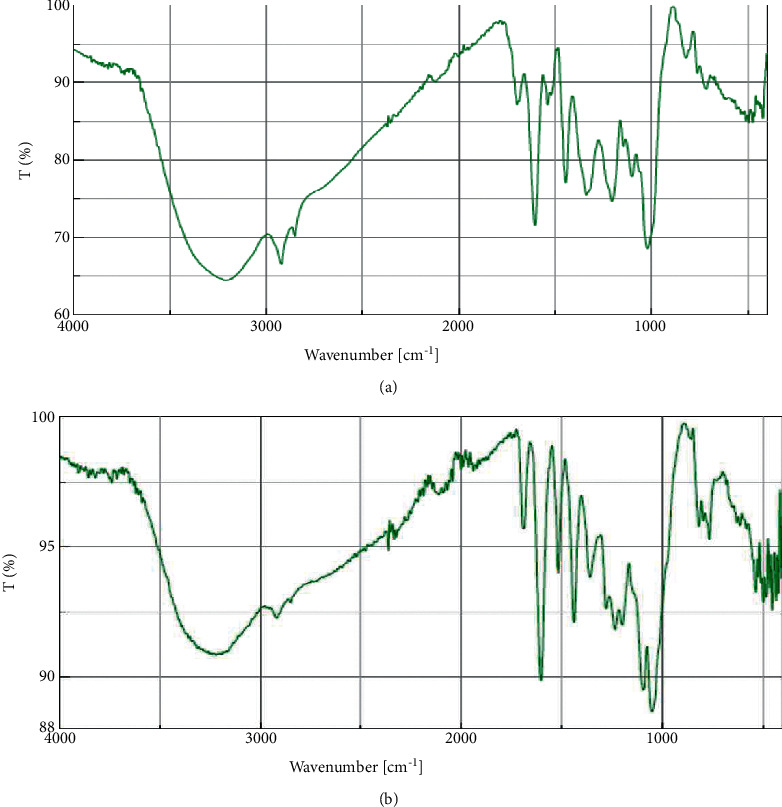
FTIR spectrum for the crude acetone (a) and methanol (b) stem bark.

FTIR analysis of the crude acetone and methanol extracts of *A. coriaria* showed peaks that corresponded to different functional groups present in the respective extract. In the acetone extracts, the peaks at 1027.87 and 1204.32 cm^−1^ are for C-O bonds, the peak at 1370.17 cm^−1^ is for C-O-H bonds, peak at 1604.48 cm^−1^ is for C=C or C=O, and peak 3208.98 cm^−1^ is for O-H (Figure 3(a)). In the methanol extracts, the peak at 1051.01 cm^−1^ is for C-O bonds, the peak at 1436.63 cm^−1^ is for C=C, the peak at 1692.23 cm^−1^ is for C=C or C=O, and the peak at 3161.72 cm^−1^ is for O–H (Figure 3(b)). All these functional groups that were identified in the FTIR analysis correspond to the different compounds identified during the GC-MS analysis (Figure 2).

### 3.5. *In Silico* Screening Results

#### 3.5.1. Docking Method Validation

**Figure 4:**
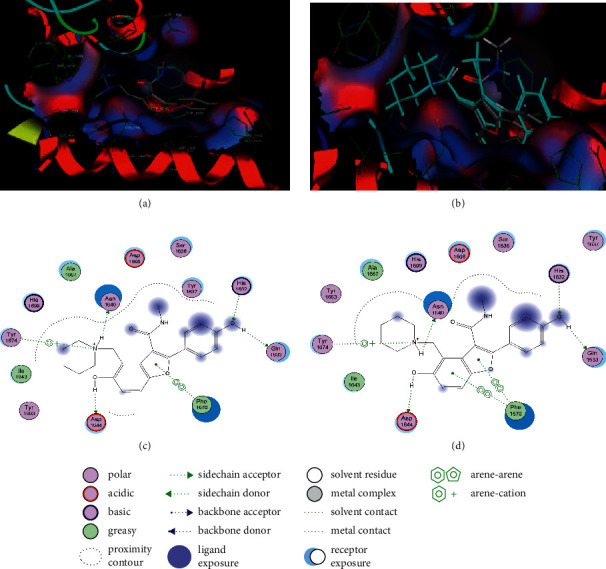
Maps of the pose for cocrystalized ligand in the binding site of polyketide synthase (a) and redocked pose of cocrystalized ligand (cyan colour) superposed on the native ligand (grey colour) (b). Interactions of cocrystalized ligand in the binding site of polyketide synthase (c). Interactions of the redocked cocrystalized ligand with amino acid residues in the binding site of polyketide synthase (d).

**Figure 5:**
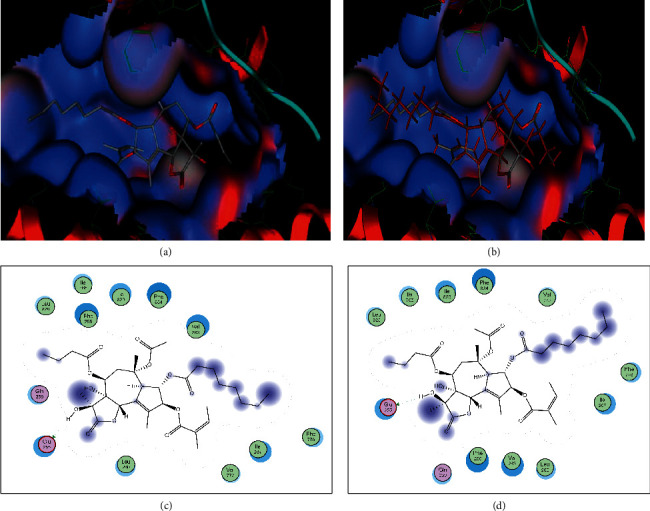
Maps of the pose of cocrystalized ligand in the binding of ATP synthase (a) and redocked pose of cocrystalized ligand (maroon colour) superposed on the native ligand (grey colour) (b). Interactions of cocrystalized ligand with amino acid residues in the binding of ATP synthase (c). Interactions of the cocrystalized ligand with amino acid residues in the binding site of ATP synthase (d).

Docking of the native ligand into the binding sites of the molecular targets resulted in root mean square deviation (RSMD) values of less than 2 Å (Table. 4). An RSMD of less than 2 Å implies that the docking method was reproducible and accurate (Figures 4 and 5).

#### 3.5.2. Binding Affinities of the Identified Compounds from *A. coriaria* for Polyketide Synthase and ATP Synthase Enzymes

**Figure 6:**
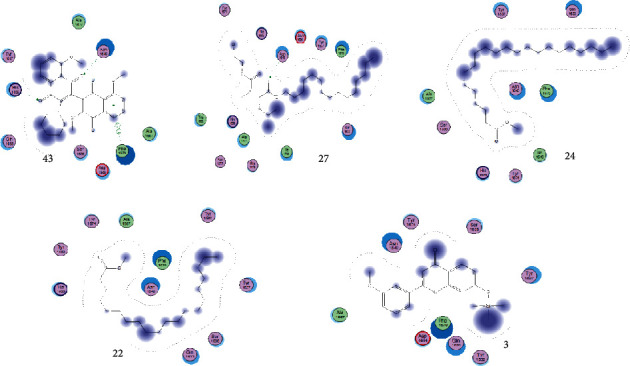
Binding interactions of the top five identified compounds with polyketide-13 synthase.

**Figure 7:**
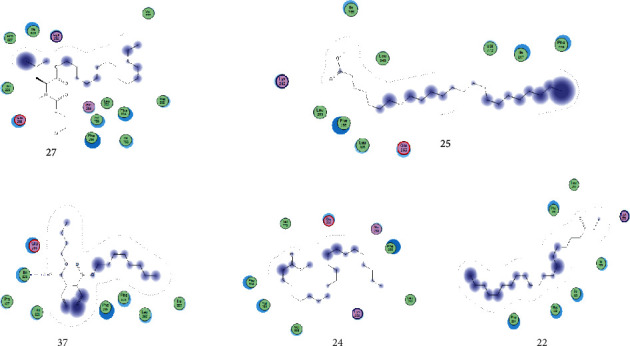
Binding interactions of the top five identified ligands with ATPsynthase.

Seven compounds identified by GC-MS analysis had better binding affinities than the two drugs (Isoniazid and rifampicin), which form the backbone of any first-line TB regimen. Five compounds had better binding affinities than bedaquiline (the standard drug known to block the mycobacterial ATP synthase enzyme) (Table 5). A visual inspection of the binding interactions of the identified compounds suggests that the ligand interacted via hydrogen bonding with amino acid Asn 1640 of the binding site of the PK13 as well as hydrophobic interactions with the amino acid residues in the binding site (Figure 6). On the other hand, visual inspection of the binding interactions of the ligands with the amino acids in the binding site of ATP synthase suggested that the ligand formed hydrophobic interactions with the close contact amino acid residues (Figure 7).

### 3.6. Descriptors of Pharmacokinetic and Toxicity

Descriptors of pharmacokinetic and toxicity properties of the top docked compounds were calculated to predict and give an insight into the drug-like properties of the compounds. These parameters can be used to guide future optimization of the promising compounds into leads by focusing on those compounds with optimum ADMET properties. Among the pharmacokinetic descriptors that were calculated included water solubility, human intestinal absorption, plasma protein binding (PPB), blood-brain barrier (BBB) penetration, CYPP450 inhibition, volume of distribution (VD), clearance, half-life, and skin permeation. On the other hand, the toxicity parameters that were calculated included AMES toxicity, hERG blockers, hepatotoxicity, and skin sensitization (Table 6).

## 4. Discussion

Methanol and acetone had significantly higher yields than hexane and chloroform because of their high and intermediate polarity that allows the extraction of more compounds with intermediate to high polarity. These results are in line with the known behavior that polar solvents extract more compounds than nonpolar solvents. The results, therefore, suggest that the stem bark of *A. coriaria* contains more polar extractable phytochemicals than their nonpolar counterparts. In a related study, ethanol, a solvent of comparable polarity to methanol had the highest yield in the extraction of phytochemicals from the leaves of *A. coriaria* [13].

### 4.1. Cytotoxicity

The National Cancer Institute classified chemicals according to their cytotoxicity as follows: IC_50_ ≤ 20 *μ*g/mL = highly cytotoxic, IC_50_ 21–200 *μ*g/mL = moderately cytotoxic, IC_50_ 201–500 *μ*g/mL = weakly cytotoxic, and IC_50_ > 501 *μ*g/mL = noncytotoxic [23]. The cytotoxicity results imply that the extracts had acceptable cytotoxicity profiles against Vero E6 since their cytotoxic concentrations were well above the cut-off limit of 500 *μ*g/mL. This probably suggests that all the stem bark extracts (hexane, chloroform, acetone, and methanol) of *A. coriaria* lack inherent toxic phytochemicals that can destroy mammalian cells at normal concentrations. In line with this study, the methanol and water stem bark extracts of *A. coriaria* were nontoxic (CC_50_ > 500 *μ*g/mL) against human embryonic lung fibroblast (HEL) cells [24]. In contrast, the stem bark ethanol and DMSO extracts of *A. coriaria* harvested in central Uganda had high cytotoxicity against the human glioblastoma cell line (U87.CD4.CXCR4) with CC_50_ of 6.4 and <4 *μ*g/mL, respectively [25]. The difference could be attributed to the fact that in the latter study, a different cell line was used, and also the plant samples were harvested from a different geographical zone, which probably could have resulted in differences in the composition and abundance of secondary metabolites. Since *A. coriaria* is used in the management of TB, which is a chronic disease that develops and heals slowly, subacute and subchronic toxicity studies are needed to determine the long-term effects of the repeated administration of the bioactive plant extracts on hematological, biochemical, and histological parameters [26].

### 4.2. Antimycobacterial Activity

The observed bioactivity of the acetone and methanol extracts implies that compounds of intermediate to high polarity are responsible for the inhibition of the growth of mycobacterial species. Although we could not ascertain the exact phytochemicals responsible for the bioactivity at this stage, it is highly plausible that the observed activity is due to compound(s) present in this plant such as triterpenes, alkaloids, and flavonoids. Acacic acid lactone, lupeol, lupenone, (+)-catechin, betulinic acid, and benzyl alcohol were previously isolated from the ethyl acetate extract of the stem bark of *A. coriaria* [12]. Additionally, flavonoids, terpenoids, and alkaloids isolated from members of the family Fabaceae have been reported to possess the antimycobacterial activity [27]. The inactivity of the hexane and chloroform extracts is because these solvents extract largely nonpolar compounds. Yet nonpolar compounds have been reported to be generally inactive against several pathogens including mycobacterium [28]. The higher antimycobacterial activity of acetone extract than the methanol extract could probably be because the acetonic extract had a wider variety of phytochemicals, moreover, in slightly higher concentrations than the methanolic extract. The inferior activity of the extracts to rifampicin could probably be attributed to the crude nature of the samples, which results in matrix interferences that reduce the actual quantity of the active ingredients at the target to cause the desired activity. It is also possible that some of the compounds in the multicomponent herbal extract could also have the antagonistic effect that lowers the antimycobacterial activity of the total crude. To the best of our knowledge, this is the first study to report the *in vitro* antimycobacterial activity of *A. coriaria*. However, other species from genus Albizia such as *A. adianthifolia* have been reported to possess antimycobacterial activity [29].

### 4.3. Phytochemical Analysis

The phytochemicals present are secondary metabolites of various pathways that occur in many plant species of family Fabaceae. Some of these phytochemicals have been reported to possess good pharmacological activities against TB and hence could be responsible for the antimycobacterial properties of this plant [13]. The diverse phytochemical composition of acetone extracts is due to its intermediate polarity that allows acetone to extract a wide range of phytochemicals of intermediate and slightly high polarity [19].

**Figure 8:**
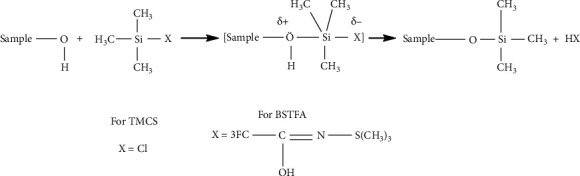
The mechanism of reaction for the derivatization of samples with BSTFA and TMCS.

Derivatization of less volatile and thermally labile phytochemicals using appropriate derivatizing agents is a method of increasing the detection of phytochemicals during GC-MS analysis. In this study, silylation using BSTFA and TMCS was used to make the phytochemicals with - OH, -COOH, =NH, -NH_2_, and -SH groups more volatile, less polar, and more thermally stable. Although BSTFA is a strong silyl donor, its donor strength is increased in the presence of TMCS [30]. The derivatizing agent displaces the active protons in the functional groups as shown in Figure 8.

All the functional groups that were identified during the FTIR analysis correlated well with the compounds identified during the GC-MS (Figure 2). The major chemical compounds identified from acetone and methanol crude stem bark extracts *A. coriaria* are secondary metabolites that form an integral part of the plants' defense systems. These secondary metabolites have been reported to have significant pharmacological properties and thus used in the management of several human and animal diseases. For example, hydroquinone, which was identified to be present in this plant was previously reported to have antimycobacterial activity with MIC of ranging between 12.5 and 50 *μ*g/mL against susceptible *M. tuberculosis* (strains H37Ra and H37Rv) and resistant strains [31]. The same compound inhibited several other clinical isolates and nontuberculous *Mycobacterium* species with MIC ranging between 12.5 to >100 *μ*g/mL and 6.125 to 25 *μ*g/mL, respectively [31]. Hydroquinone also showed antibacterial activity against extended spectrum beta-lactamase *Staphylococcus aureus, Staphylococcus aureus,* and methicillin-resistant *Staphylococcus aureus* with corresponding inhibition zones of 11, 12, and 9 mm at a concentration of 50 *μ*g/disc [32]. Hexadecanoic acid also identified to be present in this plant has been reported to have antimicrobial activity, and antioxidant, antifibrinolytic, nematicide, pesticide, 5-alpha-reductase inhibitor, anti-androgenic, hypocholesterolemia, and hemolytic properties [33]. Dibutyl phthalate has been previously reported to have the antimicrobial activity against an array of bacterial and fungal pathogens. These include *Staphylococcus epidermidis, Streptococcus pneumoniae, Escherichia coli, Micrococcus luteus, Klebsiella pneumoniae, Shigella flexneri, Vibrio cholerae,* and *P. aeruginosa* [34–37]. These bioactivities suggest that the stem bark of *A. coriaria* has the potential to provide novel molecules for the development of antimycobacterial and antibacterial agents. Apart from acting as marker compounds for bioactivity, the identified compounds can as well be used during chemical fingerprinting and profiling of chemical products especially pharmaceuticals prepared from this plant.

### 4.4. Molecular Docking Studies

In mycobacterium, polyketide-13 synthase (pks-13) catalyzes the condensation reaction (last step) in the biosynthesis of mycolic acid biosynthesis to form oxomycolic acid intermediate, which is then reduced by mycolyl reductase to form mycolic acid. Mycolic acids are long *α*-alkyl, *β*-hydroxy fatty acids consisting of 60–90 carbon atoms required for the formation of the mycobacterial cell wall and hence maintenance of cell integrity. The interactions of the mycolic acids with other cell wall components make the mycobacterial cell walls very unique and contribute to the challenges associated with mycobacterium especially its pathogenesis, persistence, and chemotherapy. Given the critical role of mycolic acids in mycobacterium cell viability and pathogenesis, enzymes involved in mycolic acids biosynthesis, such as pks-13 present novel targets for drug development [38]. On the other hand, mycobacterial ATP synthase is another validated drug target and compounds inhibiting this enzyme are promising drug candidates [39]. A unique feature of *M. tuberculosis* ATP synthase is the suppression of the ATP hydrolase activity and its inability to establish a proton gradient. This is thought to be an adaptive mechanism to prevent wastage of ATP during low oxygen conditions [40]. Bedaquiline, a newly approved antitubercular drug, binds to ATP synthase inhibiting it from releasing energy for the mycobacterial cellular activity resulting in the death of the mycobacterium [41].

The seven identified compounds that had good binding affinities for the pks-13 and ATP synthase provide insight into their potential to inhibit vital metabolic processes in the mycobacteria. For example, 3′,8,8′-trimethoxy-3-piperidyl-2,2′-binaphthalene-1,1′,4,4′-tetrone and l-leucine, *N*-(2-chloroethoxycarbonyl)-*N*-methyl-, pentadecyl ester blocked the pks-13 and ATP synthase with binding energies of -26.8626 and -28.7742 (kcal/mol). In a related study to evaluate the antimycobacterial activity of a series of 3,5-disubstituted-1,2,4-oxadiazole derivatives, pks-13 was used as a putative molecular target [38]. Like in our study, the ligands showed comparable binding affinities with the standard drugs. In contrast to our study, the main stabilizing interactions between the compounds and the enzyme were *π*-*π* interactions with the aromatic amino acid residues Phe1590, Tyr1637, Phe1585, Tyr1674, Phe1670, and the catalytic His1699 with intermittent hydrogen bonding with Ser1636 and Asn1640 [38]. In another study, five new benzofuran derivatives were reported to have sufficient binding affinity for the pks-13 synthase with an alternative binding mode to the active site [42]. All the top five ligands that inhibited the ATP synthase had at least long alkyl chains with a terminal carboxylic acid group. They therefore effectively interact with the amino acids in the catalytic site and dissipate the proton motive force (PMF), thereby decreasing the synthesis of ATP through uncoupled oxidative phosphorylation. The antimycobacterial activity of SQ109 (sequella), an antitubercular drug in phase II clinical trials, was elucidated to be due to its highly *α*-branched aliphatic moieties, which are more effective in suppressing the PMF and thus inhibit the synthesis of ATP [40]. Hydrolysis of pyrazinamide (a standard drug for TB) to pyrazinoic acid resulted in the dissipation of the PMF and hence the inhibition of ATP synthesis in *M. tuberculosis* [40]. Thus, some of the identified compounds contain carboxylic acids on long alkyl groups, and it is highly probable that the two synergistically interact with various amino acids in the catalytic site of ATP synthase and inhibit the PMF, thereby decreasing ATP biosynthesis.

### 4.5. Pharmacokinetic and Toxicity Evaluation (ADMET Descriptors)

All the compounds had acceptable physicochemical properties with acceptable violations of the Lipinski rules except for the logarithm of the n-octanol/water distribution coefficient (Log *P*), which was violated by compounds **27, 22, 3, 25,** and **37** (log *P* greater than 3). Additionally, all the compounds had moderate water solubility with their log S ranging between −4.9 and −7.8 (optimum solubility ranges between −2 and −4.0). With exception of compounds **43** and **37**, the rest had low oral gastrointestinal absorption. This implies that these compounds might need optimization to increase their water solubility and hence absorption as they need to first dissolve in water to be absorbed [22]. All the compounds were not substrates of the P-glycoproteins (P-gp). On the other hand, with exception of compounds **43**, **3,** and **37**, the rest were also not inhibitors of the P-glycoproteins. P-Glycoproteins are efflux pumps that actively pump drugs out of the cells, thereby decreasing their intracellular concentrations. They particularly protect the central nervous system from accumulating xenobiotics. Physiologically, P-gp has a fundamental role in the secretory processes [22]. All the compounds had good skin permeation except compound **43,** which had a skin permeation value of greater than −5 cm/s.

All the compounds had high plasma protein binding (greater than 90%) and hence high chances of adsorbing onto the plasma proteins. This might contribute to these compounds having a small therapeutic index and hence high chances of toxicity. The compounds also showed acceptable volumes of distribution. All the compounds had a low fraction unbound in plasma implying that there are less likely to efficiently transverse the cellular membranes and reach the target sites. All the compounds showed the inability to penetrate through the blood-brain barrier except compound **37** implying that they could not reach the brain tissues. The blood−brain barrier prevents small molecules (98%) and larger molecules (100%) from entering the central nervous system (CNS). However, it allows transport of only water- and lipid-soluble molecules and selective transport of some molecules and drugs especially those that are substrates of active transporters such as glucose and P-gp transporters [43].

Compound **3** showed inhibitory potential on all the cytochrome P450 (CYP 450) enzymes, whereas compounds **43, 27,** and **37** showed the inhibition of CYP 2C9 and CYP3A4. CYP 1A2 was inhibited by all the compounds except compound **43**. All compounds were substrates to CYP2C9, whereas compound **3** was in addition a substrate for CYP2D6 and CYP 1A62. CYP 450 enzymes are mixed functional oxidases located majorly in the liver and intestine and are responsible for the metabolism of about 60% of xenobiotics such as drugs, steroids, carcinogens, and eicosanoids. They mainly catalyze reductions, oxidation, hydrolysis, and epoxidation reactions that result in the transformation of the parent compound into either less or more toxic metabolites. Compounds that inhibit CYP450 can lead to serious drug-drug interactions that might result in toxicity or pharmacokinetic enhancement. Compounds that are substrates to CYP 450 are most likely to be metabolized, and this can result into either their deactivation or creation of more active metabolites [22].

With the exception of compounds **25** and **37**, the rest of the compounds had low clearance rates implying that they are more likely to accumulate and stay longer in than body. Clearance affects both the half-life and bioavailability of drugs, thereby directly influencing the dose and dosing frequency of a drug. As much as low clearance is advantageous as it leads to less frequent dosing, it might enhance the toxicity of the compound if the toxicity is concentration-dependent [43]. Although all the compounds were liable to cause skin sensitization, they demonstrated acceptable safety profiles on all other toxicity parameters except compound **43,** which showed mutagenic potential. In conclusion, the ADMET results imply that the top docked compounds possess variable promising pharmacokinetic properties with minimum toxicity. Hence, the most active compounds *in silico* could be isolated and considered for optimization and further validation.

### 4.6. Limitation of the Study

The NIST library could have lacked some chemical compounds that were present in the extracts and therefore could not be identified. It is also possible that some less volatile and thermally labile compounds were not derivatized and hence could not be detected by the GC-MS system.

## 5. Conclusion

There are phytochemicals in *A. coriaria* with potential antimycobacterial activity and acceptable cytotoxicity, which can be further explored and optimized for the development of new antitubercular drugs.

## Figures and Tables

**Table 1:** Extraction yield, minimum inhibitory, and cytotoxic concentrations of the extracts of *A. coriaria* stem bark.

Extract	Percentage yield (%)	Minimum inhibitory concentration (mg/mL)		Cytotoxic concentrations (*μ*g/mL)
		*M. smegmatis*	*M. tuberculosis*	Vero E6 cells
Hexane	0.48	NA	NA	>1000^a^
Chloroform	0.73	NA	NA	>1000^a^
Acetone	23.0	1250 ± 0.0^a^	937 ± 442^a^	>1000^a^
Methanol	23.2	1250 ± 0.0^a^	2500 ± 0.0^a^	>1000^a^
Rifampicin	—	15 ± 0.0	4 ± 0.0	520.02 ± 40.11

Note: ^a^*P* < 0.01 for extract vs rifampicin; NA: not active.

**Table 2:** Results of the phytochemical screening of stem bark extracts of *A. coriaria*.

Secondary metabolite	Extraction solvent			
	Hexane	Chloroform	Acetone	Methanol
Saponins	−	−	++	+
Tannins	−	−	+++	+++
Alkaloids	+	++	++	++
Coumarins	++	+	+++	+
Steroid glycosides	−	−	+	+
Triterpenes	+	+++	+++	+
Flavonoids	+	++	+++	+
Emodols	−	−	−	−
Anthocyanins	−	−	+++	++
Anthracenosides	−	−	+++	−
Reducing sugars	−	−	+++	++
Volatile oils	+++	+	+	−

Note: +++ (highly present), ++ (moderately present), + (little/traces present), and − (absent).

**Table 3:** Chemical compounds identified in the acetone and methanol extracts of *A. coriaria* stem bark using GC-MS/MS analysis.

RT (min)	Compound name	Chemical structure	Molecular formula	CAS#	Match factor	Area %
8.667	Hydroquinone (**1**)	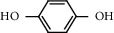	C_6_H_6_O_2_	123-31-9	71.5	10.91
9.1668	Hexanal, (2,4-dinitrophenyl)hydrazine (**2**)	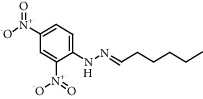	C_12_H_16_N_4_O_4_	1527-97-5	52.5	0.45
9.2805	7-Hydroxy-3′-methoxyflavone, trimethylsilyl ether (**3**)	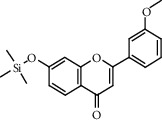	C_19_H_20_O_4_Si	1000448-98-1	52.2	3.65
10.5152	Alpha-d-glucofuranose, 6-amino-6-deoxy-1,2: 3,5-bis-O-(1-methylethylidene) (4)	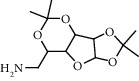	C_12_H_21_NO_5_	34322-93-5	53.8	4.41
12.6822	Octadecanoic acid, 9,10-dichloro-, methyl ester (**5**)	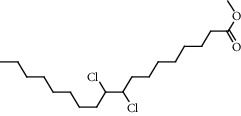	C_19_H_36_Cl_2_O_2_	33094-27-8	50.4	0.6
13.6985	3a,9b-Dimethyl-1,2,3a,4,5,9b-hexahydrocyclopenta[a]naphthalen-3-one (**6**)	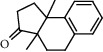	C_15_H_18_O	76803-93-5	69.2	3.29
14.4482	2,2,4-Trimethyl-1,3-pentanediol diisobutyrate (**7**)	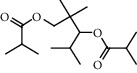	C_16_H_30_O_4_	6846-50-0	75.8	22.1
15.0766	1H-Indene, 2,3-dihydro-4,5,7-trimethyl (**8**)	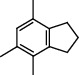	C_12_H_16_	6682-06-0	51.7	0.54
17.0349	4,5-Dihydro-3-(4-pyridinyl)-2H-benz(g)indazole (**9**)	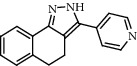	C_16_H_13_N_3_	52837-55-5	57.4	0.96
18.8234	3,3,4-Tricyano-4a-methyl-2-phenyl-2,5,6,7-tetrahydro-1-benzopyran-4-carboxamide (**10**)	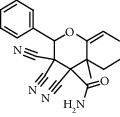	C_20_H_18_N_4_O_2_	1000459-39-9	52.1	0.98
19.5301	N,N′-Ethylenebis(2-[2-hydroxyphenyl]glycine) (**11**)	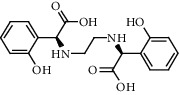	C_18_H_20_N_2_O_6_	1170-02-1	55.1	1.03
20.0803	2-Myristynoyl pantetheine (**12**)	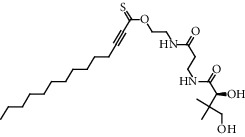	C_25_H_44_N_2_O_5_S	1000111-63-3	55.1	1.48
21.4324	1,2-Benzenedicarboxylic acid, bis(2-methylpropyl)ester (**13**)	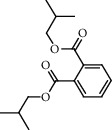	C_16_H_22_O_4_	84-69-5	66.1	3.33
22.3127	7-Hydroxy-6,9a-dimethyl-3-methylene-decahydro-azuleno [4,5-b]furan-2,9-dione (**14**)	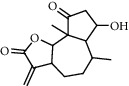	C_15_H_20_O_4_	1000296-15-9	51.9	0.29
22.833	Hexadecanoic acid, methyl ester (**15**)	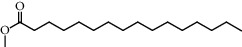	C_17_H_34_O_2_	112-39-0	88.3	49.44
23.4149	Benzenepropanoic acid, 3,5-bis(1,1-dimethylethyl)-4-hydroxy-, methyl ester (**16**)	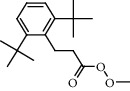	C_18_H_28_O_3_	6386-38-5	59.3	3
23.8606	1,2-Benzenedicarboxylic acid, butyl 2-methylpropyl ester (**17**)	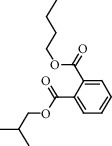	C_16_H_22_O_4_	17851-53-5	84.6	100
25.1066	Hexadecanoic acid, 14-methyl-, methyl ester (**18**)	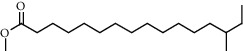	C_18_H_36_O_2_	2490-49-5	67.6	2.28
26.416	9,12-Octadecadienoic acid (Z,Z)-, methyl ester (**19**)	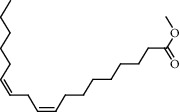	C_18_H_34_O_2_	112-63-0	87.8	46.92
26.9327	Methyl stearate (**20**)	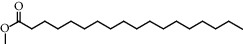	C_19_H_38_O_2_	112-61-8	82.8	21.11
28.0987	Normorphine, 2TMS derivative (**21**)	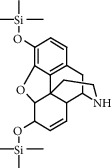	C_22_H_33_NO_3_Si_2_	55319-88-5	52.2	1.41
29.8072	Eicosanoic acid, methyl ester (**22**)	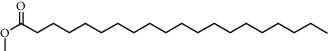	C_21_H_42_O_2_	1120-28-1	67.8	6.17
30.2999	Octadecanal, 2-bromo-(**23**)	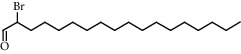	C_18_H_35_BrO	56599-95-2	60.8	1.39
32.1471	Docosanoic acid, methyl ester (**24**)	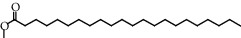	C_23_H_46_O_2_	929-77-1	67.8	5.77
34.5468	Tetracosanoic acid, methyl ester (**25**)	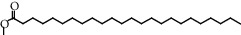	C_25_H_50_O_2_	2442-49-1	67.5	6.68
36.4072	Thiazolo [3,2-a]benzimidazol-3(2H)-one, 6,8-dimethyl-2-[[2-(trifluoromethyl)phenyl]methylidene] (**26**)	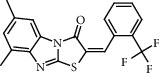	C_19_H_13_F_3_N	349497-82-1	56.2	3.06
37.1967	l-Leucine, N-(2-chloroethoxycarbonyl)-N-methyl-,pentadecyl ester (**27**)	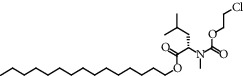	C_25_H_48_CINO_4_	1000328-52-5	50.3	0.09
38.9061	5H-Cyclohepta[b]pyridine-3-carbonitrile, 6,7,8,9-tetrahydro-2-amino-4-(4-fluorophenyl) (**28**)	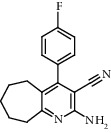	C_17_H_16_FN_3_	327098-56-6	50.5	0.2
*Methanolic extract*						
7.3899	Methyl 3-hydroxytetradecanoate (**29**)	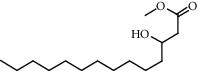	C_15_H_30_O_3_	55682-83-2	56.6	3.27
8.3156	9-(3-Fluoro-phenyl)-12-imino-10,11-dioxa-tricyclo[6.2.2.0(1,6)]dodecane-7,7,8-tricarbonitrile (**30**)	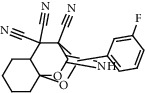	C_19_H_15_FN_4_O_2_	1000296-03-2	53.4	1.12
9.1287	2H-Pyran-3,4,5-triol, tetrahydro-2-methoxy-6-methyl (**31**)	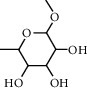	C_7_H_14_O_5_	14009-07-5	63	1.44
10.9244	Doconexent (**32**)	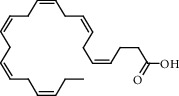	C_22_H_32_O_2_	6217-54-5	51.8	0.23
11.9629	Beta-D-Glucopyranose, 1,6-anhydro (**33**)	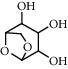	C_6_H_10_O_5_	498-07-7	71.7	14.44
12.8206	Benzoic acid, 4-(acetyloxy)-3-methoxy-, methyl ester (**34**)	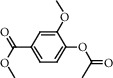	C_11_H_12_O_5_	35400-19-2	64.6	4.02
13.5814	1-Oxaspiro[4.5]decan-3-carboxylic acid, 2-oxo-4-cyano-, ethyl ester (**35**)	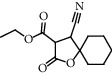	C_13_H_17_NO_4_	140650-86-8	56.5	1.01
14.454	2,2,4-Trimethyl-1,3-pentanediol diisobutyrate (**7**)	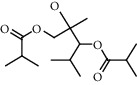	C_16_H_30_O_4_	6846-50-0	72.1	11.82
17.3793	Isoquinoline, 1-[3-methoxy-5-hydroxybenzyl]-1,2,3,4-tetrahydro-6-methoxy (**36**)	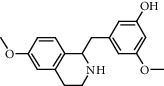	C_18_H_21_NO_3_	84230-26-2	59.5	2.76
21.4361	1,2-Benzenedicarboxylic acid, butyl octyl ester (**37**)	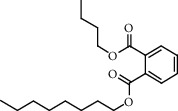	C_20_H_30_O_4_	84-78-6	56.1	0.71
22.8455	Hexadecanoic acid, methyl ester (**15**)	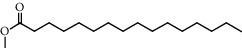	C_17_H_34_O_2_	112-39-0	85.1	38.26
23.4029	Benzenamine, N,N'-(1,2-dimethyl-1,2-ethanediylidene)bis[2,6-dimethyl (**38**)	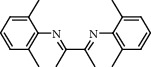	C_20_H_24_N_2_	49673-40-7	54.1	0.6
23.8634	Dibutyl phthalate (**39**)	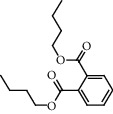	C_16_H_22_O_4_	84-74-2	82.6	46.72
25.107	Hexadecanoic acid, 14-methyl-, methyl ester (**18**)	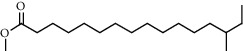	C_18_H_36_O_2_	2490-49-5	58	1.05
26.5203	9-Octadecenoic acid (Z)-, methyl ester (**40**)	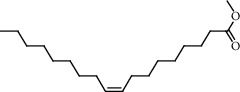	C_19_H_36_O_2_	112-62-9	89.9	100
26.938	Methyl stearate (**20**)	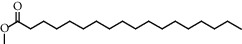	C_19_H_38_O_2_	112-61-8	84.7	15.16
27.9188	7,10,13-Eicosatrienoic acid, methyl ester (**41**)	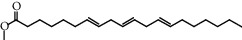	C_21_H_36_O_2_	30223-51-9	58.5	1.57
30.3004	Ethanol, 2-(9,12-octadecadienyloxy)-, (Z,Z) (**42**)	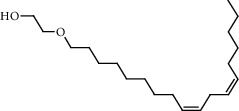	C_20_H_38_O_2_	17367-08-7	53.4	0.92
32.4456	3',8,8′-Trimethoxy-3-piperidyl-2,2′-binaphthalene-1,1′,4,4′-tetrone (**43**)	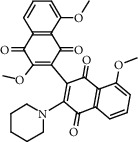	C_28_H_25_NO_7_	127611-84-1	51.9	0.86

**Table 4:** The root mean square deviation (RSMD) and binding affinities of the redocked cocrystalized ligands.

Target (PDB ID)	RMSD	Binding energy (kcal/mol)
Polyketide synthase 13 (5V3Y)	0.8288	−28.2653
ATP synthase (3A4R)	0.7076	−37.9922

**Table 5:** Top five docked ligands from those identified by GC-MS analysis on two protein targets.

SN	Polyketide synthase		ATP synthase	
	Ligand	Binding energy (kcal/mol)	Ligand	Binding energy (kcal/mol)
1	3′,8,8′-Trimethoxy-3-piperidyl-2,2′-binaphthalene-1,1′,4,4′-tetrone (**43**)	−26.8626	l-Leucine, *N*-(2-chloroethoxycarbonyl)-*N*-methyl-, pentadecyl ester (**27**)	−28.7742

2	l-Leucine, N-(2-chloroethoxycarbonyl)-N-methyl-, pentadecyl ester (**27**)	−22.1684	Tetracosanoic acid, methyl ester (**25**)	−28.3306

3	Docosanoic acid, methyl ester (**24**)	−21.8569	1,2-Benzenedicarboxylic acid, butyloctyl ester (**37**)	−26.6023

4	Eicosanoic acid, methyl ester (**22**)	−21.2508	Docosanoic acid, methyl ester (**24**)	−25.9241

5	7-Hydroxy-3′-methoxyflavone, trimethylsilyl ether (**3**)	−20.1252	Eicosanoic acid, methyl ester (**22**)	−24.0608

6	Bedaquiline	−24.0017	Bedaquiline	−22.9042

7	Isoniazid	−10.8307	Isoniazid	−11.6257

8	Rifampicin	−17.5541	Rifampicin	−18.5886

**Table 6:** Descriptors of absorption, distribution, metabolism, elimination, and toxicity (ADMET) of top docked compounds.

Properties	Compound							
	43	27	24	22	3	25	37	Acceptable values
*Absorption*								
Water solubility (log *S*)	−5.25	−7.22	−7.08	−6.47	−7.10	−7.80	−4.91	−2 to −4
GI absorption	High	Low	Low	Low	low	Low	High	High
CaCO_2_ permeability (log *P* app in 10^−6^) (cm/s)	−4.920	−4.722	−5.047	−4.964	−4.863	−5.103	−4.673	>−5.15
P-gp substrate	No	No	No	No	No	No	No	No
P-gp I inhibitor	Yes	No	No	No	Yes	No	Yes	No
Log Kp (skin permeation) (cm/s)	−6.49	−2.32	−1.22	−1.69	−5.15	−0.63	−4.13	<−5.0
F_20%_	Low	High	High	High	Low	High	High	High
F_30%_	High	High	High	High	High	High	High	High

*Distribution*								
PPB (%)	91.59	98.12	96.99	97.16	99.45	97.06	97.88	<90
BBB permeability	No	No	No	No	No	No	Yes	No
VD (L/kg)	0.430	1.265	1.577	3.112	2.906	3.814	1.367	0.04–20
Fraction unbound in plasma (%)	0.856	2.066	1.763	1.311	0.912	1.095	1.608	>5

*Metabolism*								
CYP1A2 inhibitor	No	Yes	Yes	Yes	Yes	Yes	Yes	No
CYP2C19 inhibitor	No	No	No	No	Yes	No	No	No
CYP2C9 inhibitor	Yes	Yes	No	No	Yes	No	Yes	No
CYP2D6 inhibitor	No	No	No	No	Yes	No	Yes	No
CYP3A4 inhibitor	Yes	Yes	No	No	Yes	No	No	No
CYP1A2 substrate	Yes	No	No	No	Yes	No	No	No
CYP2C19 substrate	No	No	No	No	No	No	No	No
CYP2C9 substrate	Yes	Yes	Yes	Yes	Yes	Yes	Yes	No
CYP2D6 substrate	No	No	No	No	Yes	No	No	No
CYP3A4 substrate	No	No	No	No	No	No	No	No

*Elimination*								
Total clearance (mL/min/kg)	3.78	4.35	4.63	4.65	3.41	15.96	10.38	>5
Probability that half-life is less than 3 hours	0.018	0.056	0.098	0.143	0.116	0.014	0.123	<0.3

*Toxicity*								
Carcinogenicity (AMES toxicity)	Yes	No	No	No	No	No	No	No
Cardiovascular toxicity (hERG blockers)	No	No	No	No	No	No	No	No
Hepatotoxicity	Low	No	No	No	No	No	No	No
Skin sensitization	No	Yes	Yes	Yes	Yes	Yes	Yes	No

*Physicochemical properties (Lipinski Rule #Violations)*								
Number of hydrogen bond donors (N-H, O-H): 0 to 7	Yes; 0 violation	Yes; 1 violation: log *P* > 3	Yes; 1 violation: log *P* > 3	Yes; 1 violation: log *P* > 3	Yes; 0 violation	Yes; 1 violation: log *P* > 3	Yes; 1 violation: log *P* > 3	Yes ≤ 2 violation
Number of hydrogen bond acceptors (N, O): 0 to 12								
Molecular weight: 100–600 Da								
The logarithm of the n-octanol/water distribution coefficient (log *P*): 0 to 3								

VD: volume of distribution, PPB: plasma protein binding, CL: renal clearance, F_20%_: human oral bioavailability 20%, F_30%_: human oral bioavailability 30%, and numbers correspond to the top docked compounds from Table 5. Acceptable values are according to software https://admetmesh.scbdd.com.

## Data Availability

The raw data supporting the conclusions of this study are available from the corresponding author upon request.
